# A Visual and Rapid PCR Test Strip Method for the Authentication of Sika Deer Meat (*Cervus nippon*)

**DOI:** 10.3390/ijms27010191

**Published:** 2025-12-24

**Authors:** Lijun Gao, Yuxin Xie, Yating Zhang, Yi Yang, Guangxin Yuan, Wei Xia

**Affiliations:** 1 School of Medical Technology, Beihua University, Jilin 132013, China; gaolijun@beihua.edu.cn (L.G.); 18243727597@163.com (Y.X.); 18243972517@163.com (Y.Z.); y15662150557@163.com (Y.Y.); 2 School of Pharmacy, Beihua University, Jilin 132013, China

**Keywords:** sika deer, nucleic acid test strips, DNA fingerprinting, meat products, food safety

## Abstract

The rising price of sika deer meat is increasing the risk of economic adulteration, highlighting the need for rapid and reliable authentication methods to protect both market integrity and consumers. This work presents a novel countermeasure: a polymerase chain reaction (PCR)-based nucleic acid test strip designed for the specific and visual identification of sika deer meat. Our approach commenced with the design of specific primers targeting the cytochrome C oxidase subunit I (*COI*) gene. To guarantee the reliability of the assay, a DNA standard plasmid was constructed to serve as an unambiguous positive control for the PCR. Under optimized conditions, results showed that authentic sika deer meat generated both test and control lines on the strip, while adulterated and negative samples produced only the control line. The assay demonstrated flawless specificity and a detection sensitivity of 1.0 ng·μL^−1^ for target DNA, representing a tenfold enhancement over gel electrophoresis. Furthermore, the method demonstrated a detection limit of 1% for sika deer meat in admixed samples, with a faint but visible signal observed down to 0.1% under optimized conditions. In conclusion, the developed test strip method is not only specific and sensitive but also user-friendly, positioning it as a practical and powerful tool for rapid, on-site meat authentication.

## 1. Introduction

The sika deer (*Cervus nippon*), a member of the deer family, holds significant economic and cultural value in China. It has been renowned as the “Hundred Treasures of the Whole Body” since ancient times due to the medicinal and nutritional value of its various body parts. Its antler, blood, whip, and kidney are valued as sources of rare medicinal materials in China, with believed therapeutic effects such as nourishing essence and marrow, nourishing blood and Yang, and strengthening tendons and bones [1,2,3]. The “Compendium of Materia Medica,” the classic traditional pharmacognosy text, describes sika deer meat as sweet in taste, warm in nature, non-toxic, and capable of boosting Qi, strengthening the five internal organs, and nourishing the blood and body. In addition, sika deer meat contains high protein, low fat, easy digestion, as well as various essential amino acids, trace amino acids, and rare elements for the human body, with the characteristics of easy digestion, making it a highly nutritious delicacy [4,5,6]. The increasing market value of sika deer (*Cervus nippon*) meat has led to a rise in its adulteration with lower-cost meats. This fraud undermines market fairness, consumer trust, and poses risks to public health and religious dietary practices. Current regulatory oversight and traditional authentication techniques often lack the speed, portability, and ease of use required for effective, high-throughput screening at key points in the supply chain, such as markets, warehouses, and border checks. This gap in practical enforcement tools allows fraudulent activities to persist. Therefore, there is an urgent need to develop novel, rapid, and field-deployable authentication methods specifically for sika deer meat to empower inspectors, ensure food safety, and restore integrity to the market.

At present, the identification of food mainly adopts methods such as spectrophotometry [7], HPLC [8], GC, and HPLC-MS [9,10], etc. [4]. The identification of meat products can be based on their physical and chemical properties, such as the use of liquid chromatography tandem mass spectrometry (LC-MS/MS) to accurately calculate linear correlation coefficients and recovery rates, and analyzing different species-specific peptides to accurately detect target source components [11,12]. Silva et al. used a portable near-infrared spectrometer for the quantitative analysis of beef, pork, and chicken, which could identify the adulteration in minced meat, with a good specificity and high sensitivity [13]. However, the method has shown some shortcomings such as the need for a large number of representative samples and the need for continuous updates of detection models, making the operation more cumbersome. For instance, Chen et al. [14,15] developed a sandwich ELISA using polyclonal antibodies specific to deer meat proteins, achieving a sensitive detection threshold of 0.01 μg·μL^−1^. However, immunoassays targeting proteins are of limited use for meat authentication due to potential cross-reactivity among closely related species and protein denaturation during high-temperature food processing. Genetic markers based on DNA are widely used in the identification of Chinese medicinal materials due to their ability for species-specific detection [16,17]. Polymerase chain reaction (PCR) with analysis of the resulting products by a test strip has shown good application prospects in detecting food adulteration or contamination in recent years [18,19]. For instance, Wang et al. established a PCR nucleic acid test strip for the rapid and visual identification of deer heart components [20].

This study aims to establish a rapid authentication method for sika deer meat by utilizing the species-specific DNA sequences of the cytochrome C oxidase subunit I (*COI*) gene to distinguish it from closely related species [14]. The method combines the high sensitivity of PCR with the rapid visualization of results using nucleic acid test strips, offering a reliable solution for the quality control of sika deer meat products. This study aims to establish a rapid authentication method for sika deer meat by utilizing the species-specific DNA sequences of the cytochrome C oxidase subunit I (*COI*) gene to distinguish it from closely related species. The method combines the high sensitivity of PCR with the rapid visualization of results using nucleic acid test strips, offering a reliable solution for the quality control of sika deer meat products. Therefore, this study was designed with a clear applied objective: to bridge this technological gap by developing and validating a rapid, visual, and field-deployable PCR-Lateral Flow Dipstick (LFD) assay specifically for the authentication of sika deer meat.

## 2. Results

### 2.1. Genomic DNA Extraction and Quality Verification

The alkaline denaturation method established by the previous project team was used to extract genomic DNA from animal meat samples. The A260/A280 ratios, indicative of DNA purity, ranged from 1.80 to 2.00 for all samples (Table 1). The DNA concentration variation range of the three repeated measurements was between (108.324 ± 8.124) and (359.711 ± 7.479) ng·μL^−1^. A negligible DNA concentration was detected in the blank control (0.300 ng·mL^−1^), which is attributable to instrument background signal or minimal reagent contamination. This value is at the detection limit and is over 350-fold lower than the lowest concentration measured in any meat sample. Most critically, when subjected to the specific PCR and test strip assay, the blank control yielded a negative result, demonstrating the absence of amplifiable DNA and confirming the specificity of the authentication method.

**Figure 1:**
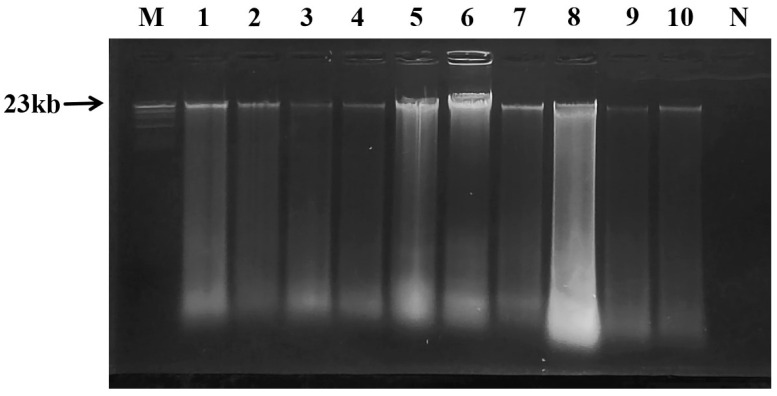
Agarose gel electrophoresis of genomic DNA of animal meat samples. M: Marker; N: negative control; 1: sika deer meat; 2: duck meat; 3: dog meat; 4: pork; 5: lamb; 6: beef; 7: chicken; 8: donkey meat; 9: rat meat; 10: mink meat.

Agarose gel electrophoresis confirmed the presence of high molecular weight genomic DNA with minimal smearing, indicating that the extracts from both sika deer and potential adulterant species were of sufficient integrity for subsequent PCR analysis (Figure 1). This verification was crucial to ensure that any negative results in the species-specific PCR assay were due to the absence of the target sequence and not to the poor quality or amplifiability of the DNA.

Rationale for Extraction Method Selection: The conventional SDS/proteinase K protocol was employed in this foundational study to ensure the consistent yield of high-purity, PCR-amplifiable DNA from all test samples, which was critical for the rigorous evaluation of the novel primer set and lateral flow detection system. It is acknowledged that this step represents the major time investment in the current workflow.

### 2.2. Design and In Silico Analysis of Species-Specific Primers

The core of this authentication method relies on the specific amplification of a DNA fragment unique to sika deer. To this end, specific primers were designed targeting the cytochrome C oxidase subunit I (*COI*) gene. The upstream primer sequence was 5′-ACA CCC TAA TCA ACT GGC-3′, and the downstream primer was 5′-AAG AAA GAA GGA GGG AGG-3′, yielding an expected product length of 526 bp.

Prior to experimental validation, the specificity of the primer pair was assessed in silico using the NCBI Primer-BLAST tool (https://www.ncbi.nlm.nih.gov/tools/primer-blast/, accessed on 21 December 2025). The computational analysis confirmed that the primers were designed to anneal specifically to the *COI* gene of *Cervus nippon* (sika deer). Crucially, no significant amplification was predicted for the genomic DNA of other common meat species, including *Sus scrofa domesticus* (pork), *Bos taurus* (beef), *Equus asinus* (donkey), *Ovis aries* (lamb), *Gallus gallus domesticus* (chicken), and *Anas platyrhynchos* (duck), among others listed in Table 1. This bioinformatic result provided strong preliminary evidence that the primer set could distinguish sika deer from its potential adulterants.

To further validate the specificity of the designed primers from a phylogenetic perspective, an in silico analysis was performed. The mitochondrial DNA sequences of several congeners, including *Cervus elaphus*, *Rangifer tarandu*, *Alces alces*, *Odocoileus hemionus* and *Cervus albirostris*, were retrieved from GenBank and aligned with the target sequence of *Cervus nippon*. The analysis revealed that the primer binding sites were perfectly complementary to the corresponding regions in *C. nippon*. In contrast, multiple nucleotide mismatches, particularly at the 3′-terminal ends critical for primer extension, were observed in all examined non-target *Cervus* species. Detailed results of the sequence alignment are provided in Supplementary Figure S1. This computational evidence strongly supports the experimental observation of no cross-reactivity and confirms the robust discriminatory power of the assay at the species level within the genus.

### 2.3. Determination of Optimal Primer Concentration and Assay Specificity

**Figure 2:**
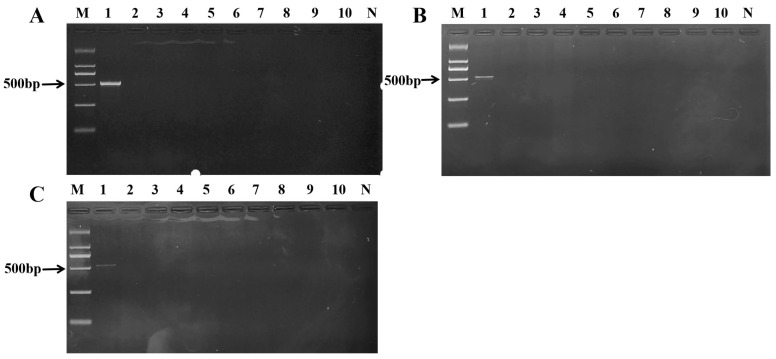
Sika deer meat primer concentration gradient detection map. (**A**) primer concentration 10.0 µmol·L^−1^; (**B**) primer concentration 5.0 µmol·L^−1^; (**C**) primer concentration 1.0 µmol·L^−1^. M: Marker; 1: sika deer meat; 2: duck meat; 3: dog meat; 4: pork; 5: lamb; 6: beef; 7: chicken; 8: donkey meat; 9: rat meat; 10: mink meat; N: negative control.

The optimal concentration of the specific primers for the PCR was first determined using sika deer DNA as the template across a concentration gradient. A clear and bright target band at 526 bp was observed across all tested concentrations, with a distinctly visible band still present at the lowest concentration of 1.0 µmol·L^−1^ (Figure 2). This concentration was therefore selected for all subsequent assays as it conserves reagents without compromising sensitivity.

Under these optimized conditions, the specificity of the assay was rigorously evaluated. PCR amplification was performed using DNA templates from a wide panel of potential adulterant species. The results demonstrated that the 526 bp amplicon was produced exclusively from sika deer DNA, including a commercially sourced regional variant (listed in Table 1). No amplification band was observed for any of the non-target species, which included pork, beef, donkey, dog, lamb, chicken, duck, rat, and mink (Figure 2).

**Figure 3:**
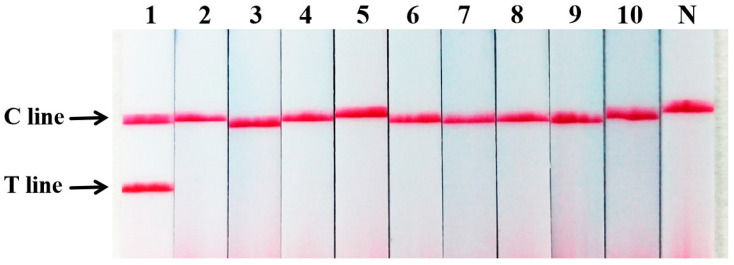
Sika deer meat PCR-nucleic acid test strip specificity test. N: negative control; 1: sika deer meat; 2: duck meat; 3: dog meat; 4: pork; 5: lamb; 6: beef; 7: chicken; 8: donkey meat; 9: rat meat; 10: mink meat.

The amplified products were further analyzed using the nucleic acid test strip for visual detection. Consistent with the gel electrophoresis results, the test strip showed two bands (Test line and Control line) only for sika deer meat samples, while all non-target meat tissues yielded only the Control line (Figure 3). The perfect agreement between the two detection methods confirms the strong specificity and reliability of the established primer set and the overall assay.

### 2.4. Construction of a DNA Standard Plasmid for Quality Control

**Figure 4:**
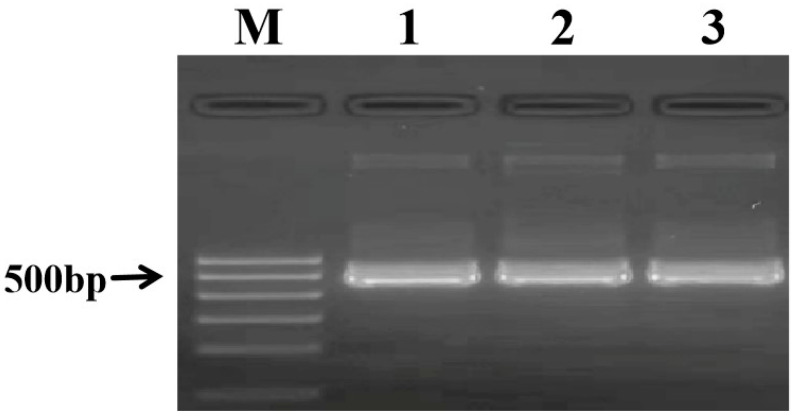
PCR verification of the recombinant plasmid. M: DNA Marker; Lanes 1–3: PCR products amplified using plasmid DNA extracted from three independent positive *E. coli* clones as the template.

To establish a reliable and consistent positive control for the assay, the specific 526 bp amplicon from sika deer was cloned and sequenced. The sequence analysis confirmed a 100% homology with the cytochrome C oxidase subunit I (*COI*) gene of *Cervus nippon*. This result unequivocally verifies that the PCR amplification produces the intended target sequence (Figure 4). The resulting plasmid was quantified and diluted to serve as a stable positive control for all subsequent PCR and test strip experiments.

### 2.5. Sensitivity of PCR Nucleic Acid Test Strip for the Detection of Sika Deer Meat

**Figure 5:**
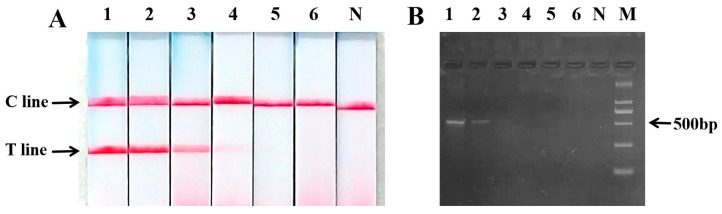
Sensitivity of PCR nucleic acid test strip for the detection of silka deer meat. (**A**) PCR-nucleic acid test strip results; (**B**) agarose gel electrophoresis graph; M: Marker; N: negative control; 1: 100 ng·μL^−1^; 2: 10 ng·μL^−1^; 3: 1 ng·μL^−1^; 4: 0.1 ng·μL^−1^; 5: 0.01 ng·μL^−1^; 6: 0.001 ng·μL^−1^.

The test results of PCR nucleic acid test strip showed that T and C lines could be still seen when the concentration of template DNA was reduced to 1 ng·μL^−1^, and the agarose gel electrophoresis showed that the target band was relatively vague when the concentration of DNA template was 10 ng·μL^−1^, indicating that the sensitivity of the PCR nucleic acid test strip was at least 10 times higher than that of agarose gel electrophoresis (Figure 5).

### 2.6. Sensitivity of PCR Nucleic Acid Test Strip for the Detection of Mixed Meat Samples

**Figure 6:**
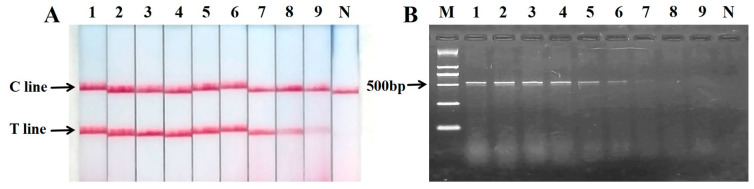
Sensitivity of PCR nucleic acid test strip for the detection of mixed meat samples. (**A**) PCR-nucleic acid test strip results; (**B**) agarose gel electrophoresis graph; M: Marker; N: negative control; 1: 100%; 2: 75%; 3: 50%; 4: 25%; 5: 10%; 6: 5%; 7: 2.5%; 8: 1%; 9: 0.1%.

The sensitivity of the assay was evaluated by testing a series of mixed meat samples with decreasing proportions of sika deer meat in chicken. The results demonstrated that the PCR nucleic acid test strip provided a clear and reliable detection limit of 1% for sika deer meat. At this concentration (Lane 8, Figure 6A), a distinct test (T) line was observed. A very faint test line was visually detectable at 0.1% (Lane 9, Figure 6A), indicating the potential limit of detection under optimal observation conditions. All sensitivity tests, including the 0.1% detection limit, were performed in triplicate (*n* = 3) experimental replicates, with 100% concordance in the visual readout.

In contrast, agarose gel electrophoresis (Figure 6B) showed a clear and reproducible band only down to 5% sika deer content (Lane 6), with a very faint band present at 1% (Lane 8). No visible band was observed at 0.1% (Lane 9). This comparison indicates that the visual readout of the nucleic acid test strip is at least fivefold more sensitive than conventional gel electrophoresis for the detection of sika deer meat in admixed samples.

## 3. Discussion

The rapid detection of adulteration in meat products is crucial for the food industry and public health [21]. In recent years, the rapid development of molecular biology has provided an emerging direction for food testing [22,23,24,25]. Compared with traditional detection methods, molecular detection technology based on genetic material markers has shown many advantages [26]. The detection method of its PCR amplification products is also constantly improving and optimizing [27]. Different from the traditional agarose gel electrophoresis, the disposable PCR nucleic acid detection strip can be used for the detection of nucleic acid amplification products due to its advantages of low cost, simplicity, rapidity, no toxicity, visualization, etc., and is gradually used for the detection of bacteria, viruses, mycotoxins, etc. [12,25,28,29,30]. For example, it is possible to quickly detect specific gene fragments of *Schistosoma japonicum*, porcine circovirus type 2 (PCV2), target derived components in mixed meat, and identify different types of animal meat by combining PCR nucleic acid test strips with recombinant enzyme-mediated nucleic acid isothermal amplification, designing primer probes, binding cross primer amplification, single plate isothermal cross primer amplification, etc., indicating that this method can be used to identify target species in raw and processed meat mixtures. Nucleic acid test strips are based on the principles of immune gold-labeled antibodies, primer labeling, and biotin signal amplification. In the detection process, the liquid sample flows in a certain direction, and the target gene amplified by the immune-labeled primer in the sample can bind to the specific immune markers on the T and C lines of the nucleic acid test strip, causing both T and C lines to produce color or optical effects. When there is no target gene amplified by the immune-labeled primer in the sample, only the C line will produce color. Compared with traditional PCR product detection methods, such as agarose gel electrophoresis, it is more rapid, convenient, and pollution-free, with a visual detection result, so it does not require high technical requirements for operators, and can be widely used in grassroots units to identify the authenticity of meat products.

As a highly nutritious delicacy, the testing of sika deer meat and its products is particularly important. The aim of this study was to establish a rapid and visual method for detecting the components of sika deer meat. From a molecular biology perspective, a specific DNA fingerprint segment for sika deer was designed by comparing and analyzing the specificity of the Cytc *COI* gene in sika deer, and its primers were labeled with biotin amplification. By optimizing the amplification conditions, the authenticity of deer meat could be identified within 30–60 s. The established three modules of DNA extraction, PCR amplification, and product detection only required the operator to add the extracted sample DNA into the PCR reaction system, and directly drop the amplification products onto the nucleic acid test strip to observe the results. It was found through the DNA extraction of cooked meat samples that the DNA extraction method established in this study had no effect on the DNA extraction of deep-processed meat products, and the detection limit for mixing different proportions of sika deer meat ingredients into chicken could be up to 0.1% by this method. The high sensitivity and simple operation of this method make it suitable for on-site rapid screening by local inspection authorities, customs, and market regulatory agencies. This technique can provide effective technical support for monitoring and combating adulteration and fraud in meat products. It should be noted that in the current workflow, the DNA extraction step using the conventional SDS/proteinase K method requires approximately three hours. While this protocol was chosen for its reliability in yielding a high-quality template for assay validation, it constitutes the primary time constraint for achieving a fully rapid, on-site workflow. For true point-of-need deployment, this step could be streamlined by adopting rapid lysis buffers (e.g., Chelex-100) or commercial field-deployable extraction kits, which could reduce the sample preparation time to under 15 min, thereby enabling a complete sample-to-answer analysis within approximately 40 min.

The high specificity of the assay was further corroborated by an in silico analysis of the primer target regions across the genus *Cervus*. Sequence alignment revealed that the designed primers exhibited perfect complementarity only to *Cervus nippon* (sika deer). In contrast, sequences from congeners such as *Cervus elaphus*, *Rangifer tarandu*, *Alces alces*, *Odocoileus hemionus* and *Cervus albirostris* showed multiple nucleotide mismatches, particularly at the 3′-terminal positions critical for polymerase extension. This genetic distinction at the primer binding sites provides a molecular explanation for the lack of cross-reactivity observed experimentally and underscores the robustness of this method in differentiating sika deer meat from other commercially relevant *Cervidae* species, which is a common scenario in meat adulteration.

While this method demonstrated perfect specificity against common domestic animals, its broader application requires careful consideration of two key parameters: phylogenetic proximity and food processing intensity. As addressed in our in silico and experimental validation, the assay effectively distinguishes sika deer from a panel of non-target species. For product applicability, the 526 bp amplicon is well-suited for the authentication of raw, fresh, frozen, and mildly processed meats, a primary target for economically motivated adulteration. In these contexts, DNA integrity is typically preserved, allowing our method to leverage its core advantages of simplicity, visual interpretation, and minimal resource requirements for rapid, on-site screening.

The assay’s performance boundaries highlight clear directions for future work. First, empirical testing against the most closely related species (e.g., *Cervus elaphus*) and across sika deer subspecies will solidify its taxonomic reliability. Second, to extend utility to the full spectrum of commercial products, including those subjected to harsh processing (e.g., retorting, prolonged high-temperature cooking), the development of a “second-generation” assay is warranted. This would involve designing new primer sets to amplify a shorter, more robust target fragment (e.g., <150 bp) within the same specific mitochondrial region validated here. Such an advancement would maintain the high specificity of the current system while ensuring sensitive detection in severely degraded DNA samples, thereby covering the entire range of food processing conditions.

Towards a Fully Integrated Field-Deployable Workflow: A key objective for translating this assay from the laboratory to point-of-use settings is the significant reduction of total processing time. The current DNA extraction procedure, while reliable, constitutes the primary bottleneck. Future development will focus on integrating the PCR-LFD detection with a rapid front-end DNA preparation step. Viable strategies include: (1) adopting simplified lysis buffers (e.g., Chelex-100 or commercially available quick-extract reagents) that require only a short incubation and centrifugation, or (2) employing portable, magnetic bead-based purification kits designed for field use. Successfully coupling a sub-15-min extraction method with the 15-min amplification/detection protocol described here would enable a complete sample-to-answer analysis in approximately 30–40 min, truly fulfilling the potential for rapid, on-site authentication of sika deer meat. (3) Prior to formal application, a thorough validation study is required to establish key performance metrics. This includes formal assessment of inter-lot and inter-operator reproducibility, calculation of false-positive and false-negative rates with confidence intervals, and statistical modeling (e.g., probit analysis) to determine the robust limit of detection (LOD) and limit of quantification (LOQ) under standardized conditions.

In conclusion, this study demonstrates the successful development of a practical, PCR-LFD-based assay for the specific detection of sika deer meat. By providing a simple, rapid, and visually interpretable tool, this work delivers a directly applicable solution for on-site screening, addressing a recognized need in the surveillance of high-value meat products. It offers a validated methodological framework that can be adopted by inspection authorities to combat food fraud and protect market integrity, underscoring the value of applied molecular research in ensuring food safety and authenticity.

## 4. Materials and Methods

### 4.1. Materials, Reagents and Instruments

Sika deer (*Cervus nippon*) meat was purchased from a local deer farm (Hongbo Deer Product Distribution Company, Shuangyang District, Changchun City, China); Pork, beef, donkey meat, dog meat, lamb, chicken, and duck meat are purchased from the local farmers’ market in Jilin City, and rat meat and mink meat were provided by the Jilin Zuojia Specialty Research Institute. The reagents and instruments used in this study are shown in Table 2.

### 4.2. Genomic DNA Extraction and Quality Identification of Animal Meats

The SDS alkaline denaturation method established by our research team before was used to extract genomic DNA from animal meat samples [14]. Firstly, the animal meat samples were chopped into small pieces, and 100 mg of the animal meat samples were weighed and placed in 1.5-mL centrifuge tubes. Five hundred μL of P1 lysis buffer (0.01 mmol·L^−1^ Tris HCl), 30 μL of P2 lysis buffer (10% SDS), and 15 μL of PK enzyme (20 mg·L^−1^ proteinase K) were added to the tubes in sequence. The sample solutions were mixed well by inverting the tubes 30 times, and the solutions were shaken in a 56 °C constant temperature water bath for 2 h (the centrifugal force was 8874× *g*). The tubes were taken out, and 500 μL of P4 lysis buffer (saturated sodium acetate solution) was added to them. The tubes were inverted 10 times to mix the solutions well, and the solutions were centrifuged at 4 °C and 8874× *g* for 8–10 min to obtain the supernatants. The supernatants were added with an equal volume of P5 (isopropanol solution), and then placed in a refrigerator at −20 °C for 40–60 min. Subsequently, the sample solutions were centrifuged at 8874× *g* for 10 min; then, the supernatant was discarded, and 500 μL of 70% ethanol aqueous solution was added to the sediment. The ethanol-sediment solution was centrifuged at 8874× *g* for 10 min, and then the sediment was rinsed 2–3 times and dried at room temperature for 40–60 min. Finally, 80 μL of TE was added to the dried sample to dissolve the DNA and obtain the animal meat genomic DNA, which should be stored at −20 °C for use. In addition, the purity and concentration of genomic DNA were detected by a micro nucleic acid protein analyzer, and the quality and integrity of genomic DNA were tested by 0.8% agarose gel electrophoresis.

### 4.3. Template DNA Amplification and Result Detection

#### 4.3.1. Primer Design

Specific primers for the sika deer *COI* gene were designed using Primer 3.0, as detailed in Section 2.2. The primers were validated using Oligo 6.0 and synthesized by Biotechnology (Changchun) Co., Ltd., Changchun, China.

#### 4.3.2. In Silico Specificity Analysis

To computationally assess the potential cross-reactivity of the primers with closely related species, an in silico specificity check was performed against representative species within the genus Cervus. The complete workflow consisted of the following steps:

Sequence Retrieval: The mitochondrial genome sequences of *Cervus nippon* and several congeneric species (including *Cervus elaphus*, *Rangifer tarandu*, *Alces alces*, *Odocoileus hemionus* and *Cervus albirostris* in public databases) were retrieved from the National Center for Biotechnology Information (NCBI) GenBank database. The accession numbers of all sequences used are listed in Supplementary Table S1.

Sequence Alignment and Primer Matching: The retrieved sequences were aligned using the Clustal Omega algorithm embedded in MEGA software (version 11.0). The forward and reverse primer sequences designed in this study were then virtually aligned against the consensus alignment to iadentify their exact binding loci.

Mismatch Analysis: Nucleotide mismatches between the primer sequences and the target binding regions of each non-target Cervus species were manually inspected and counted. Particular attention was paid to mismatches occurring within the last five nucleotides at the 3′-end of each primer, as these are known to drastically reduce or prevent polymerase extension during PCR.

Specificity Criterion: A sequence was considered unlikely to be amplified if two or more mismatches were present within the 3′-terminal five bases of either primer, or if a total of more than three mismatches were distributed across the entire primer sequence.

#### 4.3.3. PCR Amplification and Optimization of Reaction Conditions

The extracted deer meat DNA sample was diluted to a concentration of 100 ng·μL^−1^ and used as the template DNA for subsequent PCR reactions. The primer concentration gradient was explored due to the biotin amplification labeling of primers, in which the negative control was sterilized distilled water. The total PCR reaction system was 25 μL, including 12.5 μL of 2 × Taq PCR Master Mix, 0.8 μL of upstream and downstream primers (10.0 µmol·L^−1^, 5.0 µmol·L^−1^, 1.0 µmol·μL^−1^) each, and 1.5 μL of sika deer antler DNA, which were sequentially added into a 200 μL EP tube, and finally, sterilized distilled water was used to make up to 25 μL. PCR amplification procedures were pre-denatured at 95 °C for 5 min, underwent a renaturation cycle (95 °C for 30 s, 60 °C for 30 s, 72 °C for 30 s) 30 times, and extended at 72 °C for 10 min. The samples were stored at 4 °C.

#### 4.3.4. PCR Product Detection and Result Interpretation

PCR products were detected by agarose gel electrophoresis, in which the gel concentration was set to 1.5%, 3.5 μL of the nucleic acid dye GelRed was added to, the loading sample size on the electrophoresis was 10 μL and the loading sample size of the DNA molecular marker was 4 μL, the voltage was 110 V, and the electrophoresis lasted for 40–45 min. The results were observed on the ultraviolet analyzer. Simultaneously, 6 μL of PCR products were slowly dripped onto the sample area of the PCR nucleic acid reaction test strip, then the test strip was gently inserted into a centrifuge tube containing 90 μL of extending buffer, and the results could be read within 30–60 s.

### 4.4. Plasmid Construction and Sequencing

The specific 526 bp PCR product amplified from sika deer DNA was purified and cloned into the pMD19-T vector (Takara Bio, Dalian, China) according to the manufacturer’s instructions. The recombinant plasmid was transformed into *E. coli* DH5α competent cells. Positive clones were selected via blue-white screening on ampicillin-containing LB agar plates and cultured for plasmid amplification. The insertion of the target fragment was confirmed by PCR using circular plasmid DNA extracted from three randomly selected white colonies as templates (Figure 4), which ensured the reproducibility of the cloning process and excluded false positives. The plasmid from one of these confirmed clones was then purified and sent for Sanger sequencing to Sangon Biotech (Changchun) Co., Ltd., Changchun, China.

### 4.5. Sensitivity Testing

The concentration of sika deer meat DNA template was diluted to 100, 10, 1, 0.1, 0.01, and 0.001 ng·μL^−1^, respectively. The PCR amplified products were detected by using the PCR nucleic acid strip, and the test results were verified by agarose gel electrophoresis.

### 4.6. Testing of Mixed Meat Samples

Sika deer meat and chicken were cut into minced meat and mixed in proportion to make simulated mixed meat samples with different sika deer meat mass percentages (m_total_ = 100 mg). The mixed meat was cooked, and the DNA of the mixed meat was extracted to determine the minimum detection threshold of sika deer meat. The mixed meat was cooked by boiling in water at 100 °C for 15 min to simulate a moderate thermal processing. Subsequently, the DNA was extracted from the cooked mixed meat to determine the minimum detection limit of sika deer meat. The PCR amplification procedure was the same as that described in Section 4.3.2. The PCR products were detected using the nucleic acid test strip, and the final results were verified by agarose gel electrophoresis.

## 5. Conclusions

This study established a rapid detection method for sika deer meat components based on PCR nucleic acid test strips. This method offers strong specificity, rapidity, accuracy, and the ease of result interpretation, providing an effective technical approach for meat authenticity identification.

## Figures and Tables

**Table 1:** Genomic DNA concentration and purity of animal meat samples.

Sample ID	Concentration (ng·mL^−1^)	Purity (OD A260/A280)
Blank	0.300	1.000
Sika deer (*Cervus nippon*)	256.673 ± 5.046	1.850 ± 0.014
Pork (*Sus scrofa domesticus*)	163.820 ± 2.851	1.827 ± 0.012
Beef (*Bos taurus*)	172.907 ± 5.537	1.967 ± 0.025
Donkey (*Equus asinus*)	153.246 ± 2.740	1.813 ± 0.019
Dog (*Canis lupus familiaris*)	218.239 ± 2.671	1.803 ± 0.005
Lamb (*Ovis aries*)	359.711 ± 7.479	1.940 ± 0.036
Chicken (*Gallus gallus domesticus*)	112.372 ± 1.055	1.940 ± 0.022
Duck (*Anas platyrhynchos domesticus*)	108.324 ± 8.124	1.873 ± 0.009
Rat (*Rattus norvegicus*)	127.332 ± 0.902	1.833 ± 0.026
Mink (*Neovison vison*)	147.929 ± 2.101	1.976 ± 0.019

Note: The value for the blank is consistent with background signal and was confirmed to be negative in the subsequent specific PCR assay.

**Table 2:** Reagents and instruments.

Serial No.	Reagents	Instruments
1	2 × Taq PCR Master Mix	Trace Nucleic Acid UV Analyzer
	(Nanjing Nuoweizan Biotechnology Co., Ltd., Nanjing, China)	(Q6000, Thermo Fisher Scientific, Waltham, MA, USA)
2	100 bp DNA Marker	ETC 811 PCR gene amplification instrument
	(Shanghai Bioengineering Co., Ltd., Shanghai, China)	(Suzhou Dongsheng Xingye Scientific Instrument Co., Ltd., Suzhou, China)
3	DNA/HindIII, DH5α competent cells	DYY-8B Double Stable Electrophoresis Instrument
	(Beijing Dingguo Changsheng Biotechnology Co., Ltd., Beijing, China)	(Beijing Liuyi Instrument Factory, Beijing, China)
4	Agarose	High-speed refrigerated centrifuge
	(Agrose, Barcelona, Spain)	(Changsha Xiangyi Centrifuge Instrument Co., Ltd., Changsha, China)
5	Agarose gel recovery kit	UV WHITE2020D gel imaging analysis system
	(Nanjing Nuoweizan Biotechnology Co., Ltd., Nanjing, China)	(Bio-Rad, Hercules, CA, USA)
6	T-vector ligation kit and plasmid extraction kit	GeneAmp R PCR System2700 applied biosystem
	(Tiangen Biotechnology Co., Ltd., Beijing, China)	(Perkin-Elmer, Waltham, MA, USA)
7	Ampicillin (Amp)	
8	5-bromo-4-chloro-3-indolyl β-D-galactoside	
	(X-Gal) (Sigma-Aldrich, St. Louis, MO, USA)	
9	Isopropyl-beta-D-thiogalactopyranoside,	
	(IPTG) (Sigma-Aldrich, St. Louis, MO, USA)	
10	Disposable nucleic acid testing strip	
	(Hangzhou Yousida Biotechnology Co., Ltd., Hangzhou, China)	

## Data Availability

The original contributions presented in this study are included in the article/Supplementary Material. Further inquiries can be directed to the corresponding authors.

## Supplementary Materials

The following supporting information can be downloaded at: https://www.mdpi.com/article/10.3390/ijms27010191/s1.

## Abbreviations

The following abbreviations are used in this manuscript:

*COI*	Cytochrome C oxidase subunit I
PCR	Polymerase chain reaction
PCV2	Porcine circovirus type 2
ELISA	Enzyme-Linked Immunosorbent Assay
Cytc	Cytochrome c
Amp	Ampicillin
LC-MS/MS	Liquid chromatography tandem mass spectrometry

## References

[1] An L.P., Shi L.Q., Ye Y.J., Wu D.K., Ren G.K., Han X., Xu G.Y., Yuan G.X., Du P.G. (2021). Protective effect of Sika Deer bone polypeptide extract on dexamethasone-induced osteoporosis in rats. Electron. J. Biotechnol..

[2] Jin C., Cui S., Lu Y., Li Z., Huo X., Wang Y., Sha J., Sun Y. (2024). Nutritional Processing Quality of Sika Deer (*Cervus nippon*) Venison in Different Muscles. Foods.

[3] Liu K., Zhang K., Yang Y., Zong Y., He Z., Chen W., Li J., Du R. (2025). Effects of sika deer antler protein on immune regulation and intestinal microbiota in mice. J. Funct. Foods.

[4] Chen S.Q., Li Y.D., Yang Y.C., Zhao S.B., Shi H.L., Yang C.K., Wu M., Zhang A.W. (2024). Comparison of the composition, immunological activity and anti-fatigue effects of different parts in sika deer antler. Front. Pharmacol..

[5] Peng Z., Zhao H., Luo J., Sun H., Jiang Q., Zhang T. (2024). Characteristics of Meat from Farmed Sika Deer (*Cervus nippon*) and the Effects of Age and Sex on Meat Quality. Foods.

[6] Dhakal T., Jang G.-S., Kim M., Kim J.H., Park J., Lim S.-J., Park Y.-C., Lee D.-H. (2023). Habitat utilization distribution of sika deer (*Cervus nippon*). Heliyon.

[7] Li Y., Si D., Sabier M., Liu J.J., Si J.P., Zhang X.F. (2023). Guideline for screening antioxidant against lipid-peroxidation by spectrophotometer. eFood.

[8] Kulinowski L., Luca S.V., Skalicka-Wozniak K. (2023). Liquid-liquid chromatography as a promising technology in the separation of food compounds. eFood.

[9] Shrivastava A.K., Shrestha L., Pokhrel B.R., Joshi B., Lamichhane G., Vidovic B., Koirala N. (2023). LC-MS based metabolite profiling, in-vitro antioxidant and in-vivo antihyperlipidemic activity of *Nigella sativa* extract. eFood.

[10] Shi L., Wang Y., Guan Y., Men L., Sun J., Yuan G. (2025). To establish a new quality assessment method based on the regulation of intestinal microbiota in type 2 diabetes by lignans of Schisandra chinensis (Turcz.) Baill. J. Ethnopharmacol..

[11] Zhu H.X., Wei M., Zhang Y.X., Tao X.L. (2024). Analysis of Volatile Organic Compounds of Different Types of Peppers (*Capsicum annuum* L.) Using Comprehensive Two-Dimensional Gas Chromatography With Time-of-Flight Mass Spectrometry. eFood.

[12] Zhang Y.Y., Liu M.Y., Wang S.W., Kang C.D., Zhang M.Y., Li Y.Y. (2022). Identification and quantification of fox meat in meat products by liquid chromatography-tandem mass spectrometry. Food Chem..

[13] Silva L.C.R., Folli G.S., Santos L.P., Barros I., Oliveira B.G., Borghi F.T., dos Santos F.D., Filgueiras P.R., Romao W. (2020). Quantification of beef, pork, and chicken in ground meat using a portable NIR spectrometer. Vib. Spectrosc..

[14] Sun Y.H., Yue T.L., Yuan Y.H., Shi Y.H. (2023). Unlabeled fluorescence ELISA using yellow emission carbon dots for the detection of *Alicyclobacillus acidoterrestris* in apple juice. eFood.

[15] Chen X.M., Ran D., Zeng L., Xin M.G. (2020). Immunoassay of cooked wild rat meat by ELISA with a highly specific antibody targeting rat heat-resistant proteins. Food Agric. Immunol..

[16] Liu Y., Huang N. (2023). Comparison of dietary diversity and niche overlap of sympatric sika deer and roe deer based on DNA barcoding in Northeast China. Eur. J. Wildl. Res..

[17] Liu G., Luo J., Xu W., Li C., Guo L. (2023). A novel triplex real-time PCR method for the simultaneous authentication of meats and antlers from sika deer (*Cervus nippon*) and red deer (*Cervus elaphus*). J. Food Compos. Anal..

[18] Wang Y.S., Yuan G.X., Zhang L., Zhang L.H. (2023). Establishment of a multiplex-PCR detection method and development of a detection kit for five animal-derived components in edible meat. Arab. J. Chem..

[19] Gao L.J., Du B.Y., Ma Q.H., Ma Y.H., Yu W.Y., Li T., Liu Y., Yuan G.X. (2024). Multiplex-PCR method application to identify duck blood and its adulterated varieties. Food Chem..

[20] Wang Y.S., Zhang L.H., Ai J.X. (2023). Establishment of deer heart identification method and development of the detection kit based on mitochondrial cytochrome B gene. Electron. J. Biotechnol..

[21] Wang L.R., Yu X.L. (2024). Research hotspots and evolution trends of food safety risk assessment techniques and methods. eFood.

[22] Cetiz M.V., Yagi S., Kurt U., Koyuncu I., Yuksekdag O., Caprioli G., Acquaticci L., Angeloni S., Senkardes I., Zengin G. (2024). Bridging HPLC-ESI-MS/MS analysis and in vitro biological activity assay through molecular docking and network pharmacology: The example of European nettle tree (*Celtis australis* L.). eFood.

[23] Ozkan G., Sakarya F.B., Akdas A., Atalar M.N., Aydogan C., Yurt B., Capanoglu E. (2024). Comprehensive LC-MS/MS phenolic profiling of *Arum elongatum* plant and bioaccessibility of phenolics in their infusions. eFood.

[24] Wang Y.T., Niu H.M., Ma Y., Yuan G.X. (2024). Isolation, Purification, Fractionation, and Hepatoprotective Activity of Polygonatum Polysaccharides. Molecules.

[25] Niu J.-M., Cui L., Ai J.-X., Yuan G.-X., Sun L.-Y., Gao L.-J., Li M.-C. (2021). Establishment of a PCR Method for the Identification of Mink-Derived Components in Common Edible Meats. J. Anal. Test..

[26] Li Y., Wang Y., Li M., Zhang L., Yuan G.X. (2021). Development of a species-specific PCR assay for authentication of Agkistrodon acutus based on mitochondrial cytochrome b gene. Electron. J. Biotechnol..

[27] Li Z.T., Sun J.Y., Ai J.X., Li Y.N., Li M.C., Zhang L.H., Yuan G.X. (2017). Development of *Zaocys dhumnades* (Cantor) DNA test kit and its application in quality inspection of commercial products. Biomed. Res.-India.

[28] Li M.C., Gao L.J., Qu L., Sun J.Y., Yuan G.X., Xia W., Niu J.M., Fu G.L., Zhang L.H. (2016). Characteristics of PCR-SSCP and RAPD-HPCE methods for identifying authentication of *Penis et testis cervi* in Traditional Chinese Medicine based on cytochrome b gene. Mitochondrial DNA Part A.

[29] Li M.C., Wang M., Zhou Y.Q., Li Z.T., Yuan G.X., Wang X.S., Xia W., Chen J.Y. (2018). Identification and characteristics of Testudinis Carapax et Plustrum based on fingerprint profiles of mitochondrial DNA constructed by species-specific PCR and random amplified polymorphic DNA. Mitochondrial DNA Part B-Resour..

[30] Zhang X.M., Zhou T.T., Yu W.J., Ai J.X., Wang X.S., Gao L.J., Yuan G.X., Li M.C. (2018). Development and evaluation of a PCR-based assay kit for authentication of *Zaocys dhumnades* in traditional Chinese medicine. Mitochondrial DNA Part A.

