# Supplementary material for: A Visual and Rapid PCR Test Strip Method for the Authentication of Sika Deer Meat (Cervus nippon)
Source: Int J Mol Sci. 2025 Dec 24;27(1):191. doi: 10.3390/ijms27010191 (PMC12785597; doi:10.3390/ijms27010191)
Supplement: Supplementary file 1 [file ijms-27-00191-s001.zip › ijms-4032120-supplementary.pdf]

A Visual and Rapid PCR Test Strip Method for the Authentication of Sika Deer Meat (Cervus nippon)

Lijun Gao [1,†], Yuxin Xie [1,†], Yating Zhang [1], Yi Yang [1], Guangxin Yuan [2,*] and Wei Xia [1,*]

[1] School of Medical Technology, Beihua University, Jilin 132013, China; gaolijun@beihua.edu.cn (L.G.); 18243727597@163.com (Y.X.); 18243972517@163.com (Y.Z.); y15662150557@163.com (Y.Y.)

[2] School of Pharmacy, Beihua University, Jilin 132013, China

* Correspondence: bhdxygx@beihua.edu.cn (G.Y.); xiawei@beihua.edu.cn (W.X.); Tel.: +86-4324608281 (G.Y.)

† These authors contributed equally to this work.

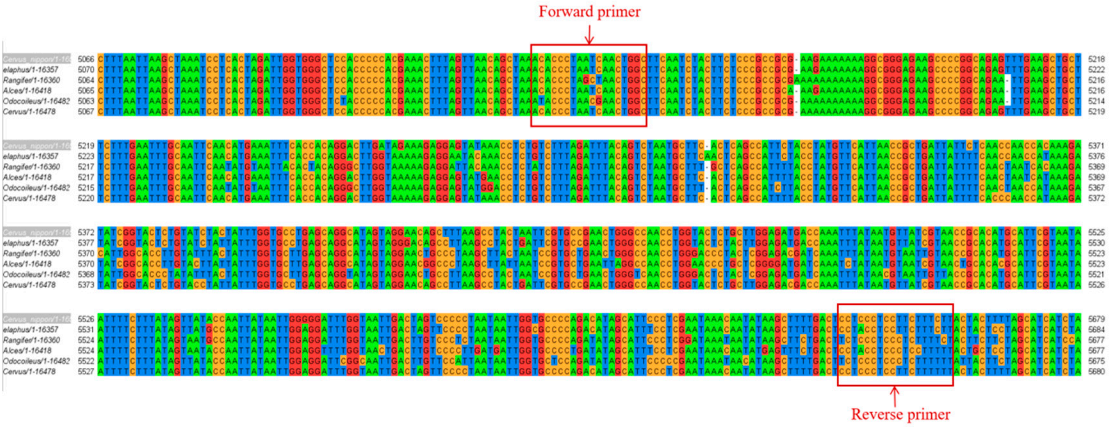

Figure S1. *In silico* analysis of primer specificity across *Cervus* species.

Table S1. Sequence information used for the *in silico* specificity analysis.

No.	Species Name	GenBank Accession No.
1	*Cervus nippon*	LC880090.1
2	*Cervus elaphus*	MF872247.1
3	*Rangifer tarandus*	MT753444.1
4	*Alces alces*	MF784604.1
5	*Odocoileus hemionus*	JN632670.1
6	*Cervus albirostris*	HM049636.1
